# miRror-Suite: decoding coordinated regulation by microRNAs

**DOI:** 10.1093/database/bau043

**Published:** 2014-06-06

**Authors:** Yitzhak Friedman, Solange Karsenty, Michal Linial

**Affiliations:** ^1^Department of Biological Chemistry, The Alexander Silberman Institute of Life Sciences, Sudarsky Center for Computational Biology and ^2^The Selim and Rachel Benin School of Computer Science and Engineering, The Hebrew University of Jerusalem, Jerusalem 91904, Israel

## Abstract

MicroRNAs (miRNAs) are short, non-coding RNAs that negatively regulate post-transcriptional mRNA levels. Recent data from cross-linking and immunoprecipitation technologies confirmed the combinatorial nature of the miRNA regulation. We present the miRror-Suite platform, developed to yield a robust and concise explanation for miRNA regulation from a large collection of differentially expressed transcripts and miRNAs. The miRror-Suite platform includes the miRror2.0 and Probability Supported Iterative miRror (PSI-miRror) tools. Researchers who performed large-scale transcriptomics or miRNA profiling experiments from cells and tissues will benefit from miRror-Suite. Our platform provides a concise, plausible explanation for the regulation of miRNAs in such complex settings. The input for miRror2.0 may include hundreds of differentially expressed genes or miRNAs. In the case of miRNAs as input, the algorithm seeks the statistically most likely set of genes regulated by this input. Alternatively, for a set of genes, the miRror algorithm seeks a collection of miRNAs that best explains their regulation. The miRror-Suite algorithm designates statistical criteria that were uniformly applied to a dozen miRNA-target prediction databases. Users select the preferred databases for predictions and numerous optional filters/parameters that restrict the search to the desired tissues, cell lines, level of expression and predictor scores. PSI-miRror is an advanced application for refining the input set by gradually enhancing the degree of pairing of the sets of miRNAs with the sets of targets. The iterations of PSI-miRror probe the interlinked nature of miRNAs and targets within cells. miRror-Suite serves experimentalists in facilitating the understanding of miRNA regulation through combinatorial– cooperative activity. The platform applies to human, mouse, rat, fly, worm and zebrafish.

**Database URL:** http://www.mirrorsuite.cs.huji.ac.il.

## Introduction

MicroRNAs (miRNAs) are short non-coding RNAs that act through base-pairing to regulate the expression of their cognate mRNAs post-transcriptionally. The RNA-induced silencing complex (RISC) complex mediates the binding of miRNAs to mRNAs. The bound miRNA-mRNA either destabilizes the mRNA or attenuates its effectiveness in translation. The outcome is monitored by mass spectrometry proteomics and by negatively regulated gene expression (1). A change in the expression of miRNAs is not confined to embryogenesis, development or cell differentiation. Instead, miRNAs govern cellular homeostasis and their expression is a hallmark of cells' status (2). Specifically, miRNAs act in stem cell differentiation, cell division, immunological cell function, organogenesis and cell identity. Moreover, miRNAs are involved in numerous pathogens, including cancer, neurodegenerative diseases and viral infection (3–5).

Currently, mirBase is the most exhaustive and comprehensive collection of miRNAs with ∼30 000 mature miRNA sequences from ∼200 organisms (6). The >2600 mature miRNAs from human and ∼1900 from mouse are estimated to target about half of the genes in humans and rodents (7). A growing number of tools and algorithms have been developed for predicting miRNA-target pairs. Over a dozen miRNA target-predicting databases have also been developed (8, 9). These tools combine features derived directly from the sequence. The most informative features are derived from the miRNA seed sequences (10). A ‘canonical’ seed has been defined as a string of seven nucleotides on the miRNA (at position 2–8). Most miRNA-targets have full base-pairing complementarity with the seed sequences.

However, the different resources incorporate additional features to different extents. These features include the binding energy, thermodynamics of the miRNA-target duplex, taxonomical conservation and the statistical context of the binding sites. More sophisticated miRNA-target predictions provide results that seek a consensus and minimal agreement from different resources. The consistency among major miRNA-target predictors is poor, reflecting the large fraction of false-positives associated with each resource (11, 12). An additional source for inconsistency among the different miRNA-target predictors originates from using Ensembl or UCSD as a source for gene lists (13). To overcome such pitfalls and to provide a functional relevance to miRNA regulation, several tools replace the pairing of miRNAs with their targets, with information on the association of miRNAs with diseases and pathways (14–16). Quantitative measures for the expression of miRNAs and mRNAs are used to filter out for a particular experiment many of the unsupported miRNA-gene predicted pairs (17).

There are a variety of algorithms that attempt to assign scores to miRNA-target pairs. Unfortunately, the reliability of the scores in view of experimental data is rather poor (12). Furthermore, substantial discrepancies remain when comparing the experimental technologies and the computational ones (18). TarBase is a reliable resource for experimentally validated miRNA-target pairs (19). miRecords is a literature-based archive (20) that extracts validated results from publications. Both resources report mostly about *in* *vitro* miRNA experiments.

The data collected from the cross-linking and immunoprecipitation (CLIP) (21, 22) and the cross-linking, ligation and sequencing of hybrids (CLASH) (23) technologies suggest that some of the assumptions of the miRNA-target pairing need to be revisited (24). Specifically, in addition to the canonical seed-pairing rule that dominated the current miRNA-target predictions, non-canonical sites (referred to as ‘nucleation bulges’) were widespread and their functionality was validated. Furthermore, the sequences of miRNA-protected regions (from CLIP and CLASH) are not limited to the 3′-UTR, but a large fraction of them were associated with the mRNA’s coding region (23).

However, the combinatorial nature of the 75 miRNA–mRNA interactions has been unequivocally confirmed (25). Specifically, it was shown that multiple miRNA-binding sites occupy the same transcript and consequently augment its regulation level. However, only 10% of the genes were regulated by a single miRNA (26).

Small-scale experiments have confirmed the synergistic effect on the transcriptional level of candidate genes by overexpressing a small number of miRNAs into cells (27). Similarly, the parallel overexpression of few miRNAs had an enhanced effect on targeted pathways (28) when compared with the effects achieved by each miRNA separately.

Our goal is to provide predictions that reflect the *in vivo* combinatorial nature of miRNAs. The predictions from miRror2.0 and Probability Supported Iterative miRror (PSI-miRror) are ranked according to the internal score (henceforth, miRIS) (29). From a set of genes that are downregulated under some experimental conditions, a coordinate action of miRNAs that regulate their expression can be anticipated. In this article, we present the rationale behind miRror-Suite and illustrate the components of this flexible platform. The miRror-Suite is composed of two application tools: miRror2.0 and PSI-miRror. The platform transforms noisy miRNA predictions from the many miRNA-target prediction databases (MDBs) into a statistically sound interpretation. PSI-miRror is an iterative protocol that aims to refine the input sets by increasing its coherence to their candidate targets at each of the iterations. The tools presented in miRror-Suite are designed to support experimentalists seeking a guideline for understanding the posttranscription regulation by miRNAs. The tools are useful for a rational design of miRNA perturbation experiments and for the elucidation of cooperative networks among miRNAs.

## Databases for mirna-Target Predictions

Database files that are included in miRror are the collection of the most stable miRNA-target prediction tools. (i) TargetScan database (30) is operated in two modes: (a) Sites that are conserved across most vertebrates and (b) site that are only conserved across mammals. All the other predictions are poorly conserved; (ii) microCosm—based on the miRanda algorithm (31); (iii) microRNA.org, which allows analysis on multiple miRNA acting on the same gene-target based on miRanda algorithm (32) (two selections for conservation is based on TargetScan family definition); (iv) PicTar (two selections for a conservation among mammals or among mammals and chicken, called 4-ways and 5-ways, respectively) (33); (v) DIANA–MicroT (34); (vi) PITA (35). PITA-Top refers to the higher confidence miRNA-target pairs in which a seed must be of a size of 7–8 nucleotides (no mismatches) with a minimal conservation. A relaxed version of all potential sites is called PITA-All (i.e. 6-mer seeds, no conservation filter); (vii) MirZ (36); (viii) miRDB resource (37); (ix) TargetRank (two selections for species’ conservation) (38) and somewhat smaller resources of (x) miRNAMap2 (39), (xi) RNA22 (40) and (xii) the meta-predictor MAMI (http://mami.med.harvard.edu/).

A total of 18 MDBs are associated with humans. Several of these resources (PITA, TargetScan, microRNA.org, TargetRank and PicTar) provide separated lists for their predictions. Each list is associated with its level of confidence, coverage and specificity. MirZ (36), microRNA.org (32) and miRBase (6) also provide information related to miRNA expression profiles for a large number of tissues and cell lines. The validated targets from TarBase (19) are not incorporated to miRror-Suite as an independent MDB but are indicated on the result output (when relevant).

The definition of miRNA family according to miRBASE is based on conservation. We set out to compress miRNAs to families solely according to the identity in the canonical seed (positions 2–8). Table 1 summarizes the list of MDBs and the number of miRNAs, genes and miRNA-families that are supported. Mapping miRNA to families led to an average compaction of 2.4 (Table 1, the ratio of the miRNAs to the number of families in each MDB). The representation of miRNAs according to their seed family reduces the redundancy in the miRNA identifiers. For example, MDBs often use different naming to the same sequence (e.g*.* hsa-miR-20b, hsa-miR-20* and hsa-miR-20-5p). By assigning all of these sequences under a unified ‘seed’ family, we gain a substantial consistency among the predictions. In miRror2.0, we allow mapping of miRNAs to their families. We encourage the user to explore this possibility in case that the size of the input is large (>100 miRNAs or genes). The miRNA family assignment reduces the number of predictions without sacrificing the significance (as determined by miRIS).

**Table 1.** miRNA-target prediction databases from miRror-Suite

MDB	Genes	miRNAs	miRNA families	Compact ratio
MAMI	11 914	317	150	2.11
PITA all	17 216	677	348	1.95
PITA top	8292	677	348	1.95
PicTar 4way	7817	178	98	1.82
PicTar 5way	2972	129	70	1.84
RNA22	11 641	313	148	2.11
TargetRank all	14 874	555	261	2.13
TargetRank conserved	10652	127	75	1.69
TargetScan conserved	25 186	1536	406	3.78
TargetScan nonconserved	30 802	1538	407	3.78
miRDB	28 890	1918	303	6.33
miRNAMap2	6040	470	260	1.81
microCosm	16 933	711	261	2.72
microRNA.org	15 400	677	348	1.95
microRNA.org conserved	31 526	248	125	1.98
microRNA.org nonconserved	32 601	850	276	3.08
microT	16 882	555	261	2.13
mirZ	31 302	1205	396	3.04

## Mapping and Identifiers’ Unification

The capacity of miRror to combine many MDBs and resources is based on converting the miRNAs and gene-targets identifiers. miRror uses the RefSeq identifier as a central entry. miRror supports UniProtKB accession and IDs, official gene symbol, Enterez ID, Flybase accession and Ensembl ID. Conversion was performed using BioMart (www.ensembl.org/biomart), ID converter (idconverter.bioinfo.cnio.es) and UniProtKB ID mapping (http://www.uniprot.org/mapping). Collectively, 15 identifiers are supported for the identifiers’ exchange among MDBs. The match of RefSeq to the original accessions of the MDBs reaches 98% for human and mouse and is lower for the other supported organisms. Currently, gene isoforms are not explicitly supported. However, most MDBs are not updated on a regular basis. Thus, the inconsistency in nomenclature remains a major challenge and a source of confusion. The collection of miRNAs and genes from all the supported MDBs that are analyzed by miRror-Suite is about 40 000 genes and 2500 miRNAs.

## Principles and Statistical Basis

The miRror platform is used to propose a minimal gene list, which is tightly regulated by a set of miRNAs. Conversely, it suggests a collection of miRNAs matching an input set of regulated genes (e.g. as resulting from a functional genomics experiment). The core of the statistical basis of miRror is the miRtegrate algorithm. The probability of the miRNAs’ interaction with the input gene set yields a calculated *P*-value threshold for all probabilities that are more significant than the indicated threshold (41). Formally, the probability of the miRNA interaction with the input gene set, as opposed to the rest of the genes in that MDB, is calculated. We applied a statistical threshold to ensure that the contribution of any MDB that covers only a small number of miRNAs with high specificity remains significant.

**Figure 1.**
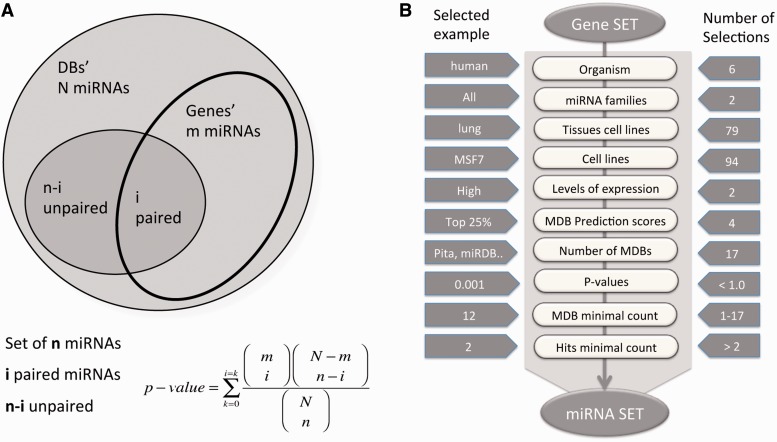
miRror2.0 statistical model and a workflow. (**A**) A schematic illustration for the hypergeometric statistic that underlies miRror is shown. For each gene, we took into account the list of miRNAs that were associated with it. We calculated the probability of the gene’s interaction with the collection of miRNAs in the user set as opposed to the rest of the miRNAs in that MDB. (**B**) A workflow of Gene2miR operational mode. The filters and parameters that can be selected by the user are shown. The number of optional values for each of the parameters and filters are indicated (right) and an example for a specific value is shown (left). For a preselected set of MDBs, statistical threshold (*P* < 0.05) and a minimal requirement of two MDBs and two hits from the query are used as the default parameters. The input for the mode of miR2Gene is the set of miRNAs that follows the same scheme.

In brief, miRtegrate calculates the probability of matches between the user gene list and the genes reported to be miRNA-targets by a set of predicting MDBs. The probability of interaction between the input gene set and every possible miRNA yields a calculated *P*-value for all probabilities that achieve a user-indicated significance threshold. A correction for multiple testing was included using the FDR procedure. Formally, the probability of the miRNA matching the input gene set is calculated. The total number of miRNA (N) and the number of paired-gene targets (m) are calculated for each gene. Figure 1A illustrates the hypergeometric calculation of the *P*-value threshold for each of the results. For each of the MDBs, the number of miRNAs and the number of gene-targets are reported (Table 1). The miRror-Suite database collects the information from all 18 MDBs (for humans) after removing any overlap among them.

## Design and Implementations

The user must select one of the six supported organisms covering human, mouse, rat, worm, fly and zebrafish. These are the most extensively studied model organisms with respect to miRNAs’ regulation. The input for miRror is a set of genes or a set of mature miRNAs. The user can activate miRror2.0 in one of the two operational modes: miR2Gene or Gene2miR. In the Gene2miR mode, a set of genes is loaded as input and all miRNAs significantly associated with the input gene list are reported; significance is based on a user-defined threshold *P*-value (the default *P*-value is <0.05). Additional constraints include the requirement to match at least two of the input genes and two MDBs as the minimal requirement for supporting each miRNA–gene interaction. A minimal agreement of MDBs and a minimal coverage were adopted to remove predictions that are based on insufficient support. The same logic applies in the miR2Gene mode.

Figure 1B illustrates the workflow for miRror2.0 application in the Gene2miR mode while emphasizing the optional filters and parameters that can be selected by the user. The operation mode is determined according to the input (i.e. set of miRNAs or genes). The range of optional values, as well as examples of valid selections are illustrated (Figure 1B). Some of the filters address the selection in view of the cellular context. Such selection is based on the precalculated collection of 79 and 94 tissues and cell lines of interest, respectively (42). Note that the statistical analysis that is performed by the miRror2.0 algorithm takes into account the actual number of genes that express in each tissue and cell line. Therefore, activating the selection for a tissue or a cell line will impact the output. An additional useful parameter allows limiting the analysis to the subset of genes whose expression is maximal. Such highly expressed subsets are selected from the absolute level of expression beyond a predetermined value (30% of top expressed genes). The selection of highly expressed genes reduces the original list of the candidate genes substantially. An additional parameter addresses the fraction of the top binding sites according to the internal scores provided by each MDB. This accounts for the top 10, 25, 50 and 100% of the genes. However, not all MDBs provide a confidence score. The user selects the relevant MDBs (18 in the case of humans but only a few for zebrafish).

While any combination of MDBs that supports the selected organism is accepted, preselected set of MDBs called ‘recommended’ or ‘minimal’ are available. Such default selections are proposed for reducing the dependency among specific MDBs (e.g. only one is selected from TargetScan_all and TargetScan_conserved). Furthermore, before or after activation the miRror2.0 search, the user may change several of the default parameters:

- The *P*-value threshold (default value is *P*-value <0.05).
- The minimal number of supporting MDBs (default value is 2).
- The minimal number of input hits (default value is 2).
- The minimal value of the miRror Internal Score (i.e. miRIS).

By changing these free parameters, the user can activate a relaxed or a strict search protocol. Balancing between an increased size of the output predictions and the high specificity of predictions is achieved by altering these optional parameters.

## Performance Tests

Assessment tests were applied for inputs of genes and miRNAs. These tests include 12 and 15 MDBs for mouse and human, respectively. The performance tests were designed to recover the experimentally validated results. Specifically, for the Gene2miR mode, we used >30 large-scale differentially expressed gene profiles that originated from miRNA overexpression experiments. We tested its success in identifying subject miRNA from the unfiltered collection of the downregulated genes. We show that miRror2.0 outperformed all tested MDBs (29).

The task for the miR2Gene mode was to recover among the top miRror predictions a specific target gene. For this test, CLIP data from starBase were used (26). Analysis of all CLIP data (i.e. the assigned reads from deep sequencing technology) showed that ∼50% of the targeted genes are associated with up to eight miRNAs and 90% of the genes are targeted by up to 35 miRNAs. For each specific gene, the collection of miRNAs that were bound was used as input for miRror2.0. We repeated the test for all the miRNA collections. We conclude that in the miR2Gene and Gene2miR modes, the ‘correct’ answer was recovered at a high success rate with respect to any of the 15 tested MDBs (29).

## Comparing MiRror and Selected MDBs Performance

**Figure 2.**
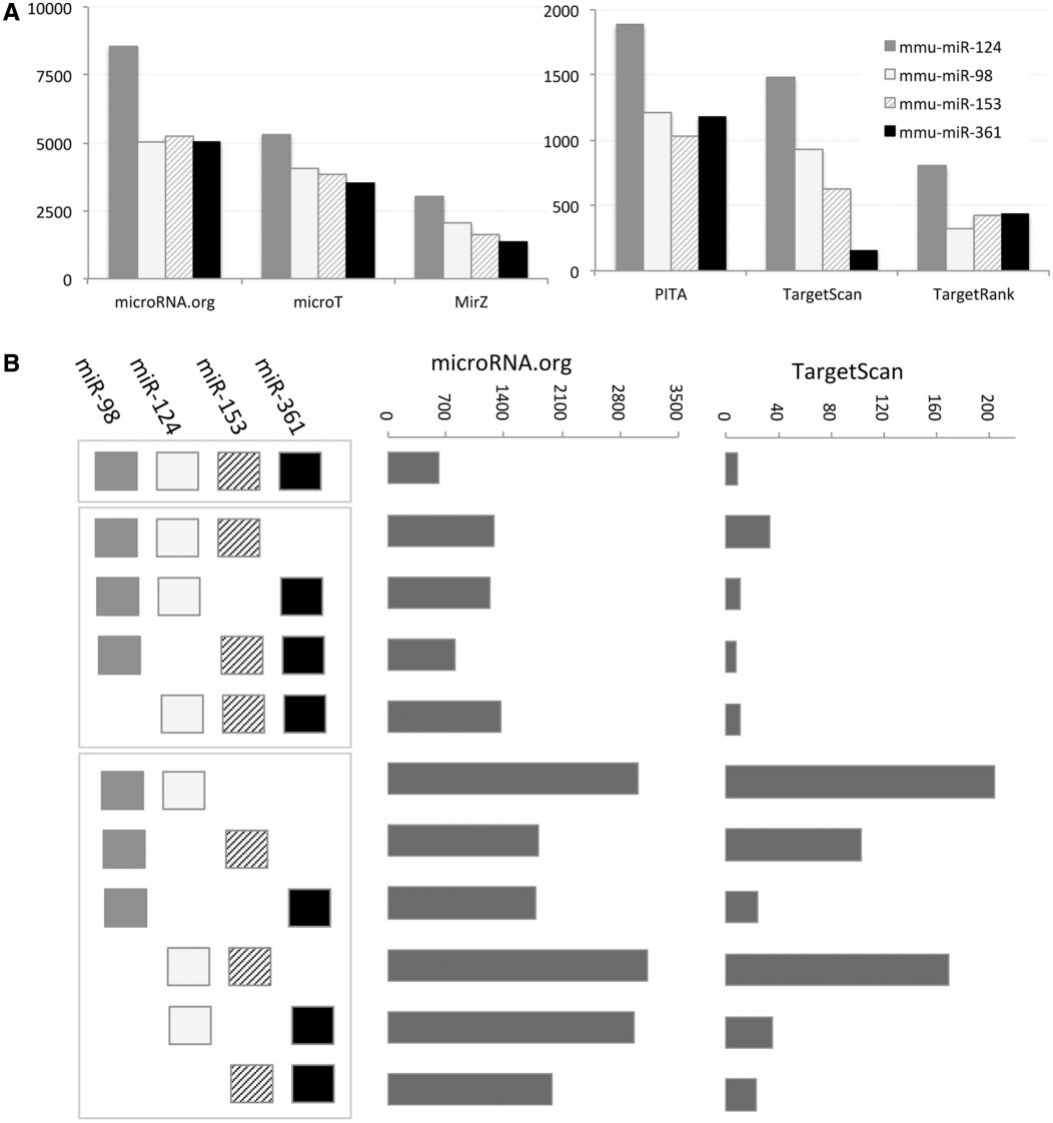
Target prediction according to six MDBs. (**A**) The number of target predicted for mmu-miR-98, mmu-miR-124, mmu-miR-153 and mmu-miR361 is shown for each of the selected MDBs: PITA, TargetScan, TargetRank, microT, miRanda (microRNA.org) and MirZ. Note the large difference in the number of targets that are reported by each MDBs. (**B**) Schematic of all possible combinations of miRNAs is shown (left). The combinations are separated to all four miRNA (one combination), three miRNAs (four combinations) and two miRNAs (six combinations). The number of predicted targets for microRNA.org is significantly higher with respect to the predictions of TargetScan.

A coordinated change in miRNAs expression is often a result of exposing cells to extreme environmental conditions (e.g. viral infection, oxidative stress and hypoxia). We illustrate the impact on the entire cell transcriptome by a simplified example in which the expression of only a few miRNAs is discussed. Figure 2A shows the difference in the mapping of the four selected mouse miRNAs (mmu-miR-124, mmu-miR-153, mmu-miR-361 and mmu-miR-98; only four miRNAs were selected for simplicity). We tested most routinely used MDBs (limited to six MDBs for simplicity). Although miRanda (microRNA.org) maps each miRNA to 5000–8000 targets, for the same set, TargetRank mapping is limited to 500–800 genes. We focus on all possible combinations among the four miRNAs for two representatives of the MDBs. There are 670 predicted targets in the intersection of all the four miRNAs according to Miranda (microRNA.org). However, there are only nine predicted targets in the intersection of these four miRNAs in TargetScan. Importantly, there are another 10 possible combinations (a combination must have at least two miRNAs). Miranda (microRNA.org) gives 1500–3000 predictions for the combinations of miRNA pairs and ∼1000 predictions for the combinations of any triple. The amount of targets for TargetScan is much lower (20–200 predictions for the miRNA-pairs and 10–30 for the miRNA-triplets, Figure 2B). This level of variability is considerably higher when all 15 MDBs are discussed. This analysis suggests that a naive application for combining the results from MDBs is unlikely to result in a useful outcome.

**Figure 3.**
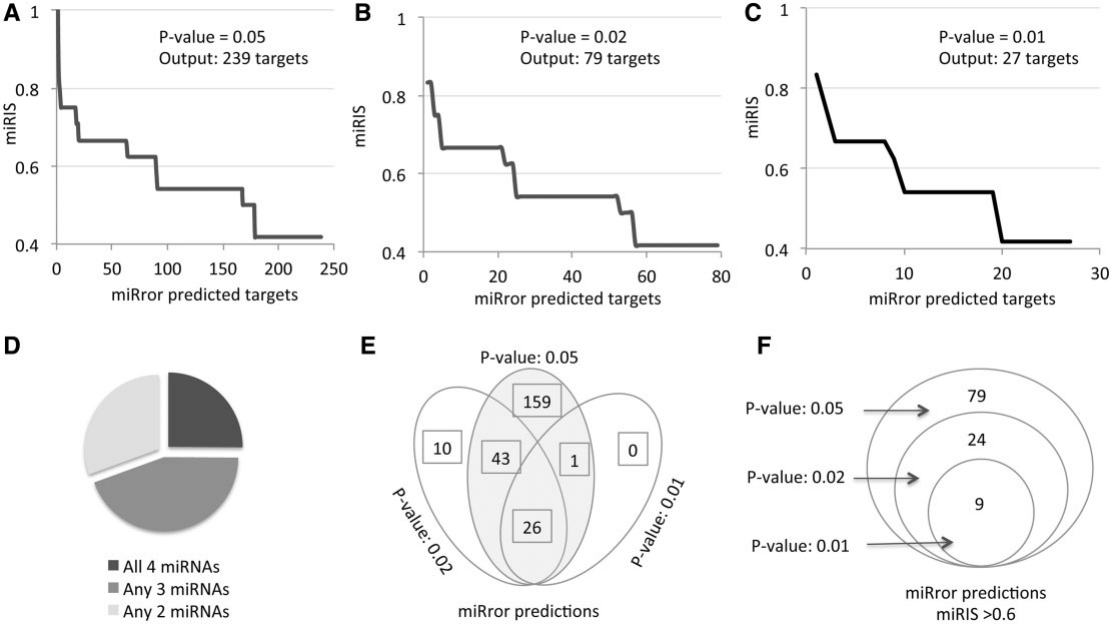
miRror2.0 prediction for a set of miRNAs. The results of miRror2.0 for the input of mmu miR-98, mmu miR-124, mmu miR-153 and mmu miR-361 are shown. The predictions are based on six MDBs (PITA, TargetScan, TargetRank, microT, microRNA.org and MirZ). The results are ranked by the values of miRIS. (**A**) Default parameters using *P*-value = 0.05. (**B**) *P*-value = 0.02. (**C**) *P*-value = 0.01. (**D**) Partition of the miRror-predicted targets according to their combination of all four, any three or any two miRNAs. The results cover the 238 predicted genes (as in A). (**E**) The stability of the results in view of changing parameters is illustrated by a Venn diagram for the three sets of results from A–C. (**F**) Venn diagram for the subset of genes shown in E, with miRIS > 0.6.

The output of activating miRror2.0 with the same set of miRNAs is shown in Figure 3. Performing the analysis by the default parameters for the six MDBs that are demonstrated in Figure 2A (PITA, TargetScan, TargetRank, microRNA.org, microT and MirZ) resulted in 239 predicted genes (Figure 3A); only 25% of them actually intersect with all four miRNAs (Figure 3D). Increasing the significance of the statistical threshold (*P*-value, Figure 1B) reduced the number of predictions substantially (for 79 and 27 predictions, respectively, Figure 3B and C). The degree of overlap among the predictions is high (Figure 3E), an indication for the stability of the approach and the validity of changing the parameters by the user.

The prediction score of miRror is called miRIS (miRror internal score). The miRIS balances between the consistency of prediction among the selected MDBs and the fraction of hits from the total size of the input, with an equal contribution of these two components:

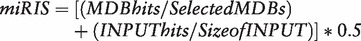

The fit between the number of MDBs that support the predictions and the ratio of the input hits from the input size is moderate (R^2 ^= 0.305). Thus, the combination of these individual measures boosts the significance of the score. Owing to the demand for a minimal number of MDBs in supporting the prediction (default = 2), miRIS is always >0. For example, in a search for 10 MDBs the minimal miRIS value is at least 0.1 (for hits that are supported by only two MDBs). Although the *P*-value is an additional quantitative estimate for each prediction, we had not included it in the miRIS score. The correlation of the predicted *P*-value {i.e. the minimal *P*-value that was reported from any of the MDBs that support such prediction, transformed to [1-log(*P*-value)]} with the fraction of MDBs or the input hits is quite significant (R^2^ = 0.496 and R^2 ^= 0.419, respectively). The user can use the *P*-value to filter the results.

We consider miRIS as a sensitive scoring method. A steep drop in miRIS (Figure 3A and C) suggests that filtration of the results by miRIS is likely to further remove noisy predictions. Indeed, when the set of predictions were limited to miRIS > 0.6, a perfect overlap in the predictions was achieved at varying statistical thresholds. The robust of the miRror2.0 application is further confirmed as using 11 instead of the six MDBs; while the miRIS value had been slightly changed, the top list of predicted genes remained identical (as shown in Figure 3F). Using miRror at a relatively restricted setting (*P*-value 0.02, miRIS >0.6, Figure 3F), result is a concise list of robust predictions that are the prime candidates for further investigation.

## Refining the MiRror Results By an Iterative Approach

The PSI-miRror is an application of miRror-Suite that provides a further refinement of the input. The principle of PSI-miRror is to iteratively search for the most likely set of miRNAs that regulates a set of genes and vice versa. Intuitively, the iterative protocol seeks a gradual improvement in the connectivity of the network that represents the miRNAs and their targeted genes.

The intuition and the usability of PSI-miRror are described below. PSI-miRror captures the concept of coordinated effect of a set of miRNAs over a gene set (as oppose to a set of miRNAs to one gene that is implemented in miRror2.0). To achieve this goal, we defined the ‘Edge-Value’ ratio (EV-ratio) that serves as an additional scoring system to the *P*-value threshold that was described for miRror. We will illustrate it for the case of miRNA as input (define as a miR2miR mode, i.e*.* the user starts with a miRNA set and the output is also a miRNA set). Given a graph of genes (G), miRNAs (M) and the edge, which represents a match between a specific miRNA and a specific gene (E), we define the EV score as the ratio between the actual number of matches divided by the maximal edges possible. 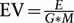.

We then combined the EV score with the miRror system through iterations. A predefined parameter determines the minimal fraction of the input (in %) that will be stable in each of the iterations. A suggested parameter for the users is 90% (and no less than 75%). The basic operation of the algorithm is greedily choose the minimal percent of M input using the EV-ratio scoring method by removing each miRNA that decreases the score. The same protocol is applied for adding an M that was not in the original input list and that improves the score. The greedy procedure was appropriate as the discussed graph of M,G is described as a bipartite graph of miRNAs and targets (i.e. no edges exists among the M or among the G). The implementation of PSI-miRror is for each MDB separately. Once the procedure was completed for all the selected MDBs and for each molecule, the output reports only the molecules that agree with the default parameter of ‘how many MDBs agree with the prediction’ (as implemented in miRror2.0).

**Figure 4.**
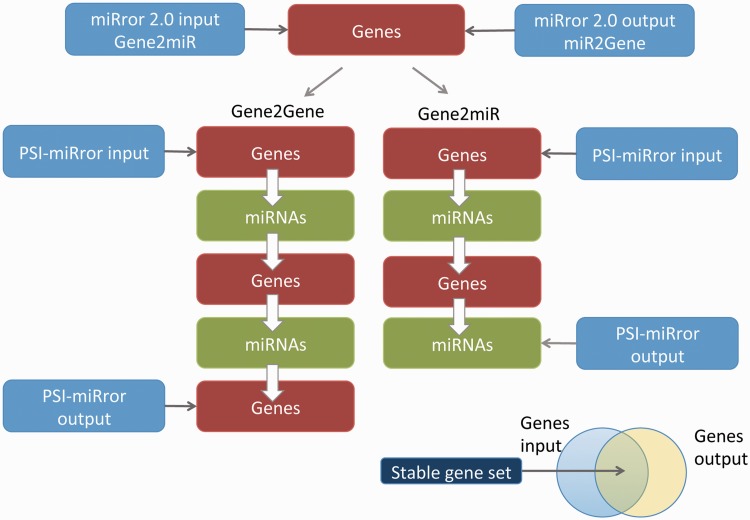
Major steps in executing the PSI-miRror application for the Gene2Gene and Gene2miR modes. The PSI-miRror input may be a list of genes from the miRror2.0 input or output. It is an iterative application that is activated in two full cycle modes (left) or in an incomplete cycle (right). The Venn diagram for the input and the output molecules is presented. The shared section represents the gene list that was unchanged during the PSI-miRror iterations. The removed genes from the input set and the added genes to the output set are listed. PSI-miRror also supports miR2Gene and miR2miR modes.

In PSI-miRror, the user must select one out of four operational modes. The ‘full iteration’ (Gene2Gene and miR2miR) starts with a set of molecules (Genes or miRNAs, respectively) and ends with a refined list of the same type of molecules as the input (Figure 4). The other two modes include the Gene2miR and miR2Gene. These are based on activating a partial iterative cycle (see scheme for Gene2miR, Figure 4). In most tested examples, the process is converged after three to four iterations.

Figure 4 illustrates full and partial cycles of the PSI-miRror application. In addition to the parameters that are used by miRror2.0, the user can select parameters that specify the iterations:

- The number of iteration (the default is set for two complete cycles).
- The relaxation level for each of the iterations (e.g. the maximal fraction of the molecules that can be removed or added per iteration).

The advantage of activating PSI-miRror is in exploring the power of ‘Set *versus* Set’ and to improve the connectivity of miRNA and targeted genes gradually using iterations. It is especially useful when in an miRNA screening experiment not all miRNAs were analyzed. In this case, the user may ask: ‘Are there additional miRNAs that could have contributed to the observed set of downregulated genes’. In a symmetrical scenario, when only partial transcriptome was measured. the user may still be interested in genes that are most likely be regulated by miRNAs. Removal of genes (or miRNAs) from the original input list signifies the genes whose connectivity to the predicted miRNA set is rather low and thus, may represent a contaminant (or a gene that is not directly regulated by miRNAs).

Importantly, the maximal fraction of the miRNAs that can be replaced in each of the iterations is predefined. Therefore, for a mode of miR2miR (input is a set of miRNAs), the resulted list of miRNAs includes, in addition to the input some miRNAs that were added (namely, were not in the input) and others that are suggested to be removed from the input. The differences between the input and the output of PSI-miRror are reported as three lists: the input, the output and the overlap list that is shared by the input and output sets (Venn diagram, Figure 4).

## Results and Visualization

**Figure 5.**
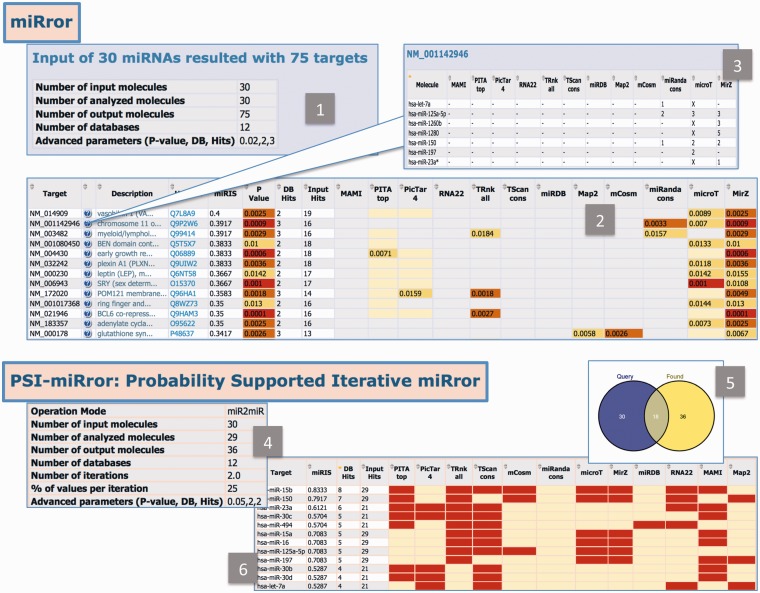
miRror2.0 and PSI-miRror results. The outputs of miRror2.0 and PSI-miRror are shown in panels 1–3 and 4–6, respectively. (1) A summary table for the miR2Gene mode. The chosen parameters and the number of analyzed molecules are listed. (2) The *P*-value thresholds are colored. The cases in which the MDB provides no prediction or a prediction that is below the selected threshold are color-coded. The table is sorted according to miRIS. (3) Zoomed table for each of the resulted gene is shown. The supported MDBs and the number of binding sites reported for each are marked. (4) PSI-miRror results in the miR2miR operational mode. (5) The Venn diagram that shows the number of genes that was included in the query as well as the number of molecules following the PSI-miRror iterations. (6) A summary table ranked by miRIS. The MDBs that contributed to the recovering of the selected genes are indicated in red.

The results from miR2Gene or Gene2miR in miRror2.0 and PSI-miRror are shown in Figure 5. Panels 1–3 illustrate the results from the miR2Gene operational mode: (i) A table that summarizes the user selections and the parameters that were applied in the search (Figure 5A, panel 1). In addition, the actual number of analyzed molecules (that may be somewhat different from the input) is reported. (ii) A main table of results in which the rows are the predicted genes. Such a table includes the genes that are associated with the maximal number of miRNA hits and the maximal number of MDBs that support the pairing of the miRNA in the input. The table is colored according to the *P*-value that is derived from the minimal value from any of the MDBs that support the predicted gene. The genes that are validated according to TarBase are highlighted (if they exist). In the main table we present the miRIS. (iii) A zoomed table (Figure 5, panel 3). For each of the resulted genes, a table is presented to indicate the actual MDBs that support the prediction. In addition, the numbers of the binding sites that are reported by each of the supported MDBs are indicated. In the current version of miRror2.0, the number of binding sites is not factored in calculating miRIS.

PSI-miRror is an operational mode that is activated on the miRror 2.0 results. The user can directly forward the miRror2.0 results to PSI-miRror. The results are presented as:

- A summarized table that shows the parameters used, the selected operational mode (i.e. any of the four modes, Figure 5, panel 4).
- A detailed table of the results, sorted by the miRIS. The supporting MDBs and number of input hits are reported.
- The PSI-miRror when activated in the Gene2Gene and miR2miR provides a Venn diagram (Figure 5, panel 5) that specifies the overlap genes (or miRNAs) that remained unchanged throughout the iterations. This sub-list should be considered as a refined list for further analysis. The molecules that were added to the input list are those that increased the overall match of the input as a set and the output as a set.

## Network View of the Results

miRror-Suite provides rich options for downloading and forwarding the results for detailed (biological) analysis. The genes’ list is forwarded to different external resources for functional annotations and pathway integration. We support the annotation enrichment schemes of DAVID (43), PANDORA (44), STRING (45) and Reactome (46). These tools allow for an investigation of the list of genes in terms of protein–protein interaction (STRING), regulatory pathways and processes (Reactome and DAVID) and association with rich functional annotations (DAVID and PANDORA). Often, the interpretation of a list of predictions is possible only at the system levels. Returning to the example described in Figures 2 and 3, and analyzing the results of miRror2.0 by a network view showed the enrichment of several functions and cell compartments. For example, by including the miRror 2.0 results (*P*-value 0.02 and the default parameters) to DAVID for annotation enrichment, several annotations stood out. The most dominant annotations are related to ‘ER, endoplasmic reticulum’ (3–6-fold change) and ‘gated channel activity’ (4–10-fold change with respect to the background of all genes). A similar enrichment was shown from the PANDORA analyses. The enriched pathways from Reactome include Calcium signaling pathway. We conclude that the tested four miRNAs from mouse (as in Figure 3) affect the ER, the ion channel activities and the calcium cell homeostasis.

## Conclusions/Future development

miRror-Suite is a platform that empowers the experimental biologists gain insights from a broad range of experimental protocols. Input for miRror2.0 and PSI-miRror can encompass miRNA profiling, global signature of proteins from mass spectrometry proteomics and lists of regulated genes from expression arrays and RNA-Seq technologies.

miRror 2.0 is based on a ‘many-to-one’ approach in which a set of miRNAs used as input is optimized for the minimal set of gene-targets that are maximally regulated by this set. Similarly, this applies to a set of genes as input. As such it is different from meta-predictors that seek consensus among alternative MDBs. miRror-Suite provides an integrative, statistically based analysis platform and presents the miRIS as a combined scoring scheme for a successful prediction of miRNA combinatorial regulations. The ‘many-to-many’ optimization is performed by the PSI-miRror approach. The user can decide on the degree of refinement relative to the original set. Activating the iterative cycles can improve the accuracy of the predictions. Varying the optional filters and parameters allows tuning the robustness and sensitivity of the results. The user may select interactively strict or relaxed parameters, and consequently control the extent of the predictions.

Currently, the platform supports six model organisms: human, mouse, rat, fly, worm and zebrafish and 18 of the largest, most stable miRNAs-target predictors. In the future, MDB predictions that consider non-canonical paring of miRNA and their targets will be included (24). Furthermore, with the increased quality and coverage of miRNAs expression, we will add a filter according to the level of miRNA expression in tissues and cell lines (17).

## References

[1] Chekulaeva M. Filipowicz W. (2009) Mechanisms of miRNA-mediated post-transcriptional regulation in animal cells. Curr. Opin. Cell Biol., 21, 452–460 19450959 10.1016/j.ceb.2009.04.009

[2] Krol J. Loedige I. Filipowicz W. (2010) The widespread regulation of microRNA biogenesis, function and decay. Nat. Rev. Genet., 11, 597–610 20661255 10.1038/nrg2843

[3] Alvarez-Garcia I. Miska E.A. (2005) MicroRNA functions in animal development and human disease. Development, 132, 4653–4662 16224045 10.1242/dev.02073

[4] Bartel D.P. (2009) MicroRNAs: target recognition and regulatory functions. Cell, 136, 215–233 19167326 10.1016/j.cell.2009.01.002 PMC3794896

[5] He L. Hannon G.J. (2004) MicroRNAs: small RNAs with a big role in gene regulation. Nat. Rev. Genet., 5, 522–531 15211354 10.1038/nrg1379

[6] Kozomara A. Griffiths-Jones S. (2010) miRBase: integrating microRNA annotation and deep-sequencing data. Nucleic Acids Res., 39, D152–D157 21037258 10.1093/nar/gkq1027 PMC3013655

[7] Bentwich I. (2005) Prediction and validation of microRNAs and their targets. FEBS Lett., 579, 5904–5910 16214134 10.1016/j.febslet.2005.09.040

[8] Rajewsky N. (2006) microRNA target predictions in animals. Nat. Genet., 38, S8–S13 16736023 10.1038/ng1798

[9] Min H. Yoon S. (2010) Got target? Computational methods for microRNA target prediction and their extension. Exp. Mol. Med., 42, 233–244 20177143 10.3858/emm.2010.42.4.032 PMC2859323

[10] Shin C. Nam J.W. Farh K.K. (2010) Expanding the microRNA targeting code: functional sites with centered pairing. Mol. Cell, 38, 789–802 20620952 10.1016/j.molcel.2010.06.005 PMC2942757

[11] Marin R.M. Vanicek J. (2011) Efficient use of accessibility in microRNA target prediction. Nucleic Acids Res., 39, 19–29 20805242 10.1093/nar/gkq768 PMC3017612

[12] Thomas M. Lieberman J. Lal A. (2010) Desperately seeking microRNA targets. Nat. Struct. Mol. Biol., 17, 1169–1174 20924405 10.1038/nsmb.1921

[13] Ritchie W. Flamant S. Rasko J.E. (2009) Predicting microRNA targets and functions: traps for the unwary. Nat. Methods, 6, 397–398 19478799 10.1038/nmeth0609-397

[14] Cho S. Jun Y. Lee S. (2011) miRGator v2.0: an integrated system for functional investigation of microRNAs. Nucleic Acids Res., 39, D158–D162 21062822 10.1093/nar/gkq1094 PMC3013691

[15] Backes C. Meese E. Lenhof H.P. (2010) A dictionary on microRNAs and their putative target pathways. Nucleic Acids Res., 38, 4476–4486 20299343 10.1093/nar/gkq167 PMC2910047

[16] Papadopoulos G.L. Alexiou P. Maragkakis M. (2009) DIANA-mirPath: Integrating human and mouse microRNAs in pathways. Bioinformatics, 25, 1991–1993 19435746 10.1093/bioinformatics/btp299

[17] Muniategui A. Pey J. Planes F.J. (2013) Joint analysis of miRNA and mRNA expression data. Brief Bioinform., 14, 263–278 22692086 10.1093/bib/bbs028

[18] Berezikov E. Robine N. Samsonova A. (2011) Deep annotation of Drosophila melanogaster microRNAs yields insights into their processing, modification, and emergence. Genome Res., 21, 203–215 21177969 10.1101/gr.116657.110 PMC3032924

[19] Vergoulis T. Vlachos I.S. Alexiou P. (2012) TarBase 6.0: capturing the exponential growth of miRNA targets with experimental support. Nucleic Acids Res., 40, D222–D229 22135297 10.1093/nar/gkr1161 PMC3245116

[20] Xiao F. Zuo Z. Cai G. (2009) miRecords: an integrated resource for microRNA-target interactions. Nucleic Acids Res., 37, D105–D110 18996891 10.1093/nar/gkn851 PMC2686554

[21] Alexiou P. Maragkakis M. Papadopoulos G.L. (2009) Lost in translation: an assessment and perspective for computational microRNA target identification. Bioinformatics, 25, 3049–3055 19789267 10.1093/bioinformatics/btp565

[22] Hafner M. Lianoglou S. Tuschl T. (2012) Genome-wide identification of miRNA targets by PAR-CLIP. Methods, 58, 94–105 22926237 10.1016/j.ymeth.2012.08.006 PMC3508682

[23] Helwak A. Kudla G. Dudnakova T. (2013) Mapping the human miRNA interactome by CLASH reveals frequent noncanonical binding. Cell, 153, 654–665 23622248 10.1016/j.cell.2013.03.043 PMC3650559

[24] Kim K.K. Ham J. Chi S.W. (2013) miRTCat: a comprehensive map of human and mouse microRNA target sites including non-canonical nucleation bulges. Bioinformatics, 29, 1898–1899 23709495 10.1093/bioinformatics/btt296

[25] Chi S.W. Zang J.B. Mele A. (2009) Argonaute HITS-CLIP decodes microRNA-mRNA interaction maps. Nature, 460, 479–486 19536157 10.1038/nature08170 PMC2733940

[26] Yang J.H. Li J.H. Shao P. (2011) starBase: a database for exploring microRNA-mRNA interaction maps from Argonaute CLIP-Seq and Degradome-Seq data. Nucleic Acids Res., 39, D202–D209 21037263 10.1093/nar/gkq1056 PMC3013664

[27] Ivanovska I. Cleary M.A. (2008) Combinatorial microRNAs Working together to make a difference. Cell Cycle, 7, 3137–3142 18927506 10.4161/cc.7.20.6923

[28] Zhou Y. Ferguson J. Chang J.T. (2007) Inter- and intra-combinatorial regulation by transcription factors and microRNAs. BMC Genomics, 8, 396 17971223 10.1186/1471-2164-8-396 PMC2206040

[29] Balaga O. Friedman Y. Linial M. (2012) Toward a combinatorial nature of microRNA regulation in human cells. Nucleic Acids Res., 40, 9404–9416 22904063 10.1093/nar/gks759 PMC3479204

[30] Grimson A. Farh K.K. Johnston W.K.*.* (2007) MicroRNA targeting specificity in mammals: determinants beyond seed pairing. Mol. Cell, 27, 91–105 17612493 10.1016/j.molcel.2007.06.017 PMC3800283

[31] John B. Enright A.J. Aravin A. (2004) Human MicroRNA targets. PLoS Biol., 2, e363 15502875 10.1371/journal.pbio.0020363 PMC521178

[32] Betel D. Wilson M. Gabow A. (2008) The microRNA.org resource: targets and expression. Nucleic Acids Res., 36, D149–D153 18158296 10.1093/nar/gkm995 PMC2238905

[33] Krek A. Grun D. Poy M.N. (2005) Combinatorial microRNA target predictions. Nat. Genet., 37, 495–500 15806104 10.1038/ng1536

[34] Maragkakis M. Reczko M. Simossis V.A. (2009) DIANA-microT web server: elucidating microRNA functions through target prediction. Nucleic Acids Res., 37, W273–W276 19406924 10.1093/nar/gkp292 PMC2703977

[35] Kertesz M. Iovino N. Unnerstall U. (2007) The role of site accessibility in microRNA target recognition. Nat. Genet., 39, 1278–1284 17893677 10.1038/ng2135

[36] Hausser J. Berninger P. Rodak C.*.* (2009) MirZ: an integrated microRNA expression atlas and target prediction resource. Nucleic Acids Res., 37, W266–W272 19468042 10.1093/nar/gkp412 PMC2703880

[37] Wang X. (2008) miRDB: a microRNA target prediction and functional annotation database with a wiki interface. RNA, 14, 1012–1017 18426918 10.1261/rna.965408 PMC2390791

[38] Nielsen C.B. Shomron N. Sandberg R. (2007) Determinants of targeting by endogenous and exogenous microRNAs and siRNAs. RNA, 13, 1894–1910 17872505 10.1261/rna.768207 PMC2040081

[39] Hsu S.-D. Chu C.-H. Tsou A.-P. (2008) miRNAMap 2.0: genomic maps of microRNAs in metazoan genomes. Nucleic Acids Res., 36, D165–D169 18029362 10.1093/nar/gkm1012 PMC2238982

[40] Miranda K.C. Huynh T. Tay Y. (2006) A pattern-based method for the identification of MicroRNA binding sites and their corresponding heteroduplexes. Cell, 126, 1203–1217 16990141 10.1016/j.cell.2006.07.031

[41] Friedman Y. Naamati G. Linial M. (2010) MiRror: a combinatorial analysis web tool for ensembles of microRNAs and their targets. Bioinformatics, 26, 1920–1921 20529892 10.1093/bioinformatics/btq298

[42] Kapushesky M. Emam I. Holloway E. (2010) Gene expression atlas at the European bioinformatics institute. Nucleic Acids Res., 38, D690–D698 19906730 10.1093/nar/gkp936 PMC2808905

[43] Huang da W. Sherman B.T. Tan Q. (2007) The DAVID Gene Functional Classification Tool: a novel biological module-centric algorithm to functionally analyze large gene lists. Genome Biol., 8, R183 17784955 10.1186/gb-2007-8-9-r183 PMC2375021

[44] Rappoport N. Fromer M. Schweiger R. (2010) PANDORA: analysis of protein and peptide sets through the hierarchical integration of annotations. Nucleic Acids Res., 38, W84–W89 20444873 10.1093/nar/gkq320 PMC2896089

[45] Jensen L.J. Kuhn M. Stark M. (2009) STRING 8–a global view on proteins and their functional interactions in 630 organisms. Nucleic Acids Res., 37, D412–D416 18940858 10.1093/nar/gkn760 PMC2686466

[46] Vastrik I. D'Eustachio P. Schmidt E.*.* (2007) Reactome: a knowledge base of biologic pathways and processes. Genome Biol., 8, R39 17367534 10.1186/gb-2007-8-3-r39 PMC1868929

